# Health status of returning refugees, internally displaced persons, and the host community in a post-conflict district in northern Sri Lanka: a cross-sectional survey

**DOI:** 10.1186/s13031-018-0176-7

**Published:** 2018-10-01

**Authors:** Rachel Burns, Kolitha Wickramage, Anwar Musah, Chesmal Siriwardhana, Francesco Checchi

**Affiliations:** 1 Faculty of Infectious and Tropical Diseases, London School of Hygiene and Tropical Medicine, London, UK; 2 International Organisation for Migration (UN Migration Agency), Sri Lanka Country Mission, 62 Ananda Coomaraswamy Mawatha, Colombo, 00300 Sri Lanka; 3 Faculty of Epidemiology and Population Health, London School of Hygiene and Tropical Medicine, London, UK

**Keywords:** Sri Lanka, Post-conflict, Mental health, Quality of life, Chronic disease, Noncommunicable diseases, Forced displacement

## Abstract

**Background:**

Although the adverse impacts of conflict-driven displacement on health are well-documented, less is known about how health status and associated risk factors differ according to displacement experience. This study quantifies health status and quality of life among returning refugees, internally displaced persons, and the host community in a post-conflict district in Northern Sri Lanka, and explores associated risk factors.

**Methods:**

We analysed data collected through a household survey (*n* = 570) in Vavuniya district, Sri Lanka. The effect of displacement status and other risk factors on perceived quality of life as estimated from the 36-item Short Form Questionnaire, mental health status from 9-item Patient Health Questionnaire, and self-reported chronic disease status were examined using univariable analyses and multivariable regressions.

**Results:**

We found strong evidence that perceived quality of life was significantly lower for internally displaced persons than for the host community and returning refugees, after adjusting for covariates. Both mental health status and chronic disease status did not vary remarkably among the groups, suggesting that other risk factors might be more important determinants of these outcomes.

**Conclusions:**

Our study provides important insights into the overall health and well-being of the different displaced sub-populations in a post-conflict setting. Findings reinforce existing evidence on the relationship between displacement and health but also highlight gaps in research on the long-term health effects of prolonged displacement. Understanding the heterogeneity of conflict-affected populations has important implications for effective and equitable humanitarian service delivery in a post-conflict setting.

## Background

### Conflict-driven displacement and health

Displacement associated with conflict is increasingly recognised as an important issue in global health. Evidence shows that conflict-driven displacement has profound impacts on both the physical and mental health of those displaced [1–3]. There are two broad categories of forced migrants: internally displaced persons (IDPs, those who remain within their national border) and refugees (those who cross an internationally recognised national border). While both groups may flee for similar root causes, their experiences with displacement and subsequent health needs are heterogeneous and multidimensional [2].

Infectious diseases and neonatal disorders remain the primary causes of excess mortality in low income, low life expectancy conflict settings [4]. However, in the past decade non-communicable diseases (NCDs) and mental health disorders have become more prominent in the research landscape due to their recognition as important sources of mortality and morbidity in conflict-affected displaced populations [5–7]. It is particularly challenging to address these distinct health problems in post-conflict settings, where recovering health systems may lack the capacity and human resources to effectively address the specific health needs of affected people [5, 8].

The indirect public health consequences of conflict do not manifest uniformly across affected populations. Research shows IDPs tend to experience higher levels of mortality and morbidity than refugees [2, 9]. Hence, understanding the unique health status and associated risk factors for sub-populations with distinct displacement profiles is vital for effective and equitable humanitarian service delivery. While many studies investigate displacement and specific health outcomes, less is known about how physical and mental health status varies within a post-conflict setting following prolonged periods of contrasting displacement experiences.

### Sri Lankan context

Due to almost thirty years of protracted internal conflict between the Liberation Tigers of Tamil Eelam and the Sri Lankan armed forces, Sri Lanka has experienced high levels of internal and external population displacement. It is estimated that almost 1 million people were displaced at the peak of the conflict in 2001, with 115,000 still internally displaced, 73,000 living in 112 camps as refugees and 34,000 additional refugees outside camps in the southern Indian state of Tamil Nadu at the end of the conflict [8, 10]. Following peace negotiations in May 2009, IDPs in camps within Sri Lanka and refugees in India started to resettle previous conflict areas, ending for some a few generations of displacement [11]. There is a growing field of research on the health of conflict-affected populations, but still a lacuna of information on the effect of protracted conflicts on health [8, 12]. As such, the displaced populations of this context provide a unique setting to investigate the long-term social, cultural, and health effects of displacement. Several studies examining the physical and mental health status of different displaced populations within conflict-affected districts of Sri Lanka have been published [8, 10, 13–15]. However, a comparison between IDP, returning refugees, and non-displaced host community members within the same geographic location has not been previously reported, to our knowledge. The objective of this study was to explore how displacement experience and other risk factors may influence mental health status, chronic disease status, and quality of life in Sri Lankan men and women living in Vavuniya district.

## Methods

### Study design and participants

The cross-sectional survey was carried out in the northern district of Vavuniya, Sri Lanka from August 2011 to October 2011. The inclusion criteria for the three sub-populations were as follows: i) Returning Refugees (RET): any household containing refugees who had returned to Vavuniya district from 1st January 2010 to 30th March 2011; ii) Internally Displaced Persons (IDP): living in Cheddikulum IDP camp, Vavuniya District at the time of the survey; iii) Host Community (HOC):never displaced internally and never sought asylum as refugee at any time over the past 10 years. Respondents were the oldest member of the household available at the time of the survey.

### Sampling

According to the United Nations High Commissioner for Refugees, the total number of refugees whose return to the district was facilitated from January 1, 2010 to March 30, 2011 was 766 persons living in 190 households. All these households were sampled. An equal number of IDP and resident households was also sampled using a simple random sampling design, using the Cheddikulum IDP camp registry for the IDP population and government household registries for the host community as the sampling frames. A total of 570 households (190 returning refugee households, 190 IDP household units, and 190 host community households) were thus included in the survey. The sample size was not powered to detect specific effects, but would, for alpha = 0.05, have been sufficient to estimate a 50% prevalence of depression (see below) within each group with precision ≤5%, and any mean quality of life score (see below) with precision ≤5% assuming a standard deviation of 20.

### Data collection and entry

The questionnaires were administered by a field research team comprised of two medical officers and eight trained graduate students under the supervision of the principal investigator (KW). Interviews were undertaken in the respondents’ home in a quiet and private space as guided by the respondent. The questionnaires were reviewed by the principle investigator for completeness and consistency, before they were manually edited and coded to prepare for data entry by the field research team. Double data entry was conducted by two members of the field research team and carried out using a programme based on MS-Access. SPSS 12.0 Command Syntax Reference 2003 was used for data tabulation and Stata 13 (StataCorp. 2013. *Stata Statistical Software: Release 13*. College Station, TX: StataCorp LP.) for data analysis.

### Outcomes

Quality of life was measured using the 36-item Short Form Questionnaire (SF-36), a multipurpose, 36-item survey that measures eight domains of health: physical functioning, role limitations due to physical health, bodily pain, general health perceptions, vitality, social functioning, role limitations due to emotional problems, and mental health. The items are transformed into a score from 0 to 100 with a higher score indicating better health [16, 17]. Previous studies support the cross-cultural validity and reliability of the SF-36 within both displaced populations [18, 19] and Sri Lanka [20, 21]. Mental health status was measured using the 9-item Patient Health Questionnaire (PHQ-9), which consists of nine questions on depression symptoms over the last 2 weeks, with responses of 0 = *not at all*, 1 = *several days*, 2 = *more than half the days*, and 3 = *nearly every day*. The item scores are summed to produce a total score range of 0 to 27. For most analyses, the PHQ-9 total score is divided into the following categories of increasing severity: 0–4 = *none*, 5–9 = *mild*, 10–14 = *moderate*, 15–19 = *moderately severe*, and 20–27 = *severe* [22]. In this study, the variable was re-categorized into presence of any depression (yes/no) as all participants but one scored in the ‘none’ or ‘mild’ categories. The PHQ-9 has been cross-culturally validated and used in post-conflict Sri Lanka [23]. Chronic disease status was based on self-report by the respondent (at least one of the following reported: hypertension, angina, heart attack, congestive heart disease, diabetes, lung diseases, cancer, mental health disorders, or chronic renal disease).

### Exposures of interest

**Fig. 1:**
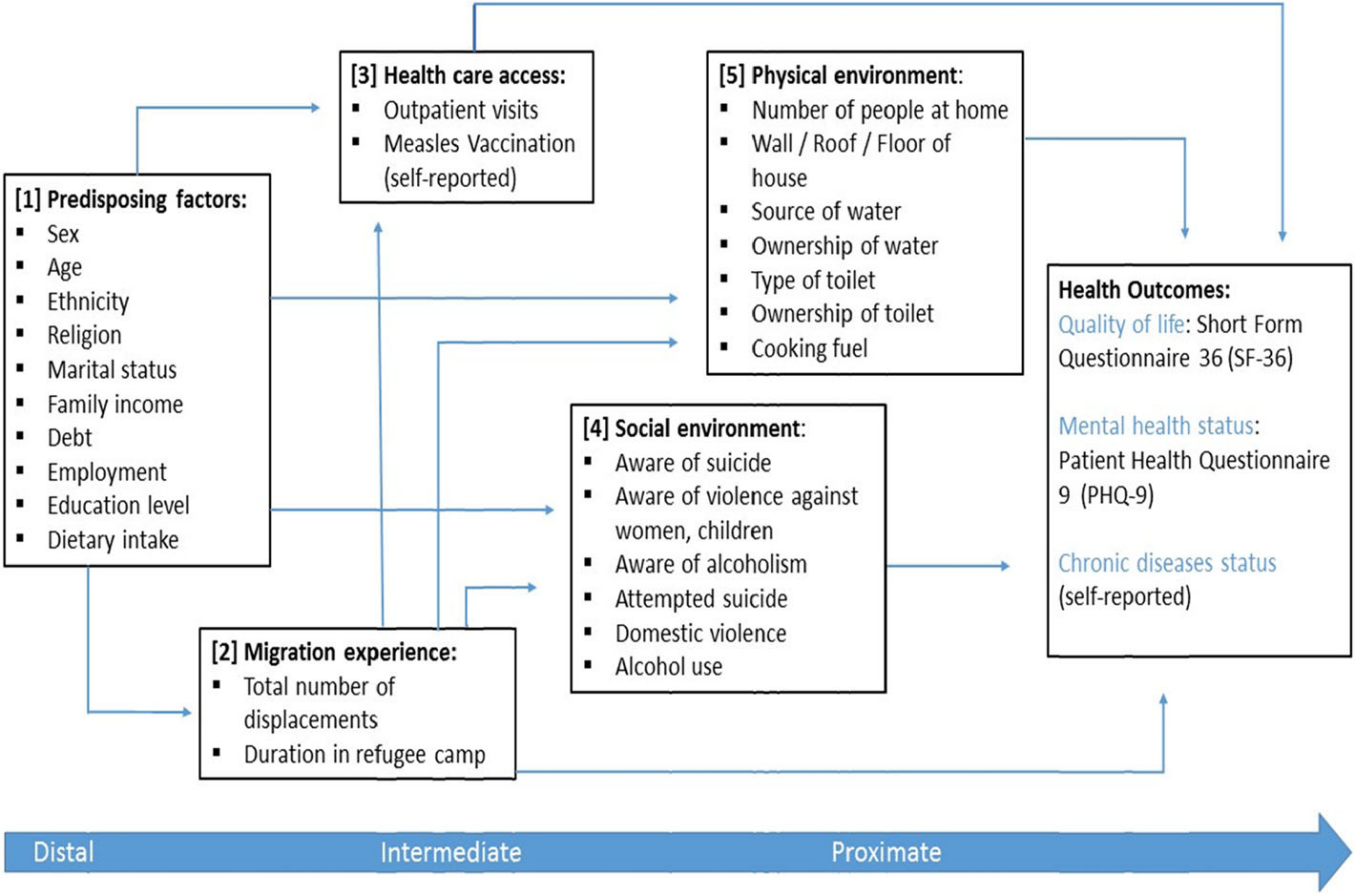
Conceptual framework showing causal relationships between the different domains of questionnaire variables and the measured health outcomes

The primary exposure of interest was the displacement status of each household. Other exposure variables were grouped into domains and organised according to a conceptual framework depicting the plausible relationships between domains of risk factors and their relationship, direct or indirect (i.e. mediated through other factors), with the main outcomes of interest (Fig. 1) [24].

### Risk factor analysis

The analysis was performed using Stata, Release 14 (Stata Corp., College Station, Texas, USA). Univariable associations of displacement status and potential risk factors with each outcome were observed. All variables that were associated (*p* < 0.15) with each outcome were included in subsequent multivariable analysis, in addition to the primary exposure (displacement status) and a priori variables sex and age,. The value of the threshold was chosen prior to the analysis to reduce the chances of incorrectly excluding potentially important risk factors. Multivariable logistic regressions were performed for mental health status and chronic disease status. A generalised linear regression with a Gaussian distribution and identity link function was employed for SF-36 total score, and effect sizes were reported as the β coefficient; robust standard errors were computed to account for the skewed distribution of SF-36 score.

For each outcome, models were constructed using the domains (numbered 1 through 5) as described in the conceptual framework (Fig. 1). First, a base model containing displacement status and a priori variables (age and sex) was fitted. Then, variables within domain 1 (predisposing factors) as retained from the univariable analysis were added, one by one, and the one that most improved model fit (based on a likelihood ratio test, LRT) or showed the smallest *p*-value for association with the outcome was added to form a new base model. The process was repeated using remaining variables within domain 1, ultimately leaving out any that did not alter the model’s effect estimates by at least 10% or had a non-significant LRT. The updated base model was then carried into the next causally downstream framework domain (domain 2), and the above process was repeated with domain 2 variables, continuing thus until all domains were exhausted [24, 25]. Lastly, to investigate any plausible effect modifications of the variables in each final model on the association between displacement status and each outcome, we fit an interaction term between the covariate in question and displacement status adjusting for the other covariates. Only significant interactions were retained in the model (*p* < 0.05).

## Results

A total of 570 households were visited (190 HOC households, 190 IDP households, and 190 RET households). If the head of the household was not present during time of visit nor following a return visit, another household was randomly selected from the relevant registry. There were 29 absentees (5%) and no refusals to participate.

The main socio-demographic characteristics, stratified by displacement status, are shown in Table 1. The majority of the participants were women (69.5%), Sri Lankan Tamil (94.6%), Hindu (76.1%) and married (79.3%). The mean age was 40.6 years and most had an average monthly family income < 1000 Sri Lankan Rupees (Rs.) (70%), were without debt (75.6%), and unemployed (73.6%). More than half of the participants had never been displaced or had only been displaced once (56.3%).

**Table 1:** Sample Socio-demographic Characteristics by Displacement Status, Vavuniya District

Variables of Interest	Total (*n* = 570)	Displacement Status N (%)			*P* value*
		HOC	IDP	RET	
		(*n* = 190)	(n = 190)	(n = 190)	
Predisposing Factors					
Sex					
Male	174 (30.5)	68 (35.8)	19 (10.0)	87 (45.8)	< 0.001
Female	396 (69.5)	122 (64.2)	171 (90.0)	103 (54.2)	
Age					
< 29	135 (23.7)	50 (26.3)	43 (22.6)	42 (22.1)	0.26
30–39	165 (29.0)	53 (27.9)	50 (26.3)	62 (32.6)	
40–49	118 (20.7)	42 (22.1)	36 (19.0)	40 (21.1)	
50–59	84 (14.7)	29 (15.3)	28 (14.7)	27 (14.2)	
> =60	68 (11.9)	16 (8.4)	33 (17.4)	19 (10.0)	
Marital Status					
Married	452 (79.3)	138 (72.6)	142 (74.7)	172 (90.5)	< 0.001
Not Married	118 (20.7)	52 (27.4)	48 (25.3)	18 (9.5)	
Ethnicity					
Sri Lankan Tamil	539 (94.6)	163 (85.8)	189 (99.5)	187 (98.4)	< 0.001
Indian Tamil/Muslim	31 (5.4)	27 (14.2)	1 (0.5)	3 (1.6)	
Religion					
Hindu	434 (76.1)	136 (71.6)	133 (70.0)	165 (86.8)	< 0.001
Other	136 (23.9)	54 (28.4)	57 (30.0)	25 (13.2)	
Monthly Family Income					
< 5000	275 (48.2)	33 (17.7)	162 (85.3)	80 (42.1)	< 0.001
5000–9999	124 (21.8)	38 (20.3)	23 (12.1)	63 (33.2)	
> =10,000	168 (29.5)	116 (62.0)	5 (2.6)	47 (24.7)	
Missing	3 (0.5)				
Debt					
Yes	139 (24.4)	5 4(28.4)	35 (18.4)	50 (26.3)	0.057
No	431 (75.6)	136 (71.6)	155 (81.6)	140 (73.7)	
Employment Status					
Yes	149 (26.1)	57 (30.0)	10 (5.3)	82 (43.2)	< 0.001
No	421 (73.9)	133 (70.0)	180 (94.7)	108 (56.8)	
Education Level					
No school	38 (6.7)	6 (3.2)	20 (11.5)	12 (6.3)	< 0.001
Grade 1 to 5	117 (20.5)	28 (14.7)	45 (23.7)	44 (23.2)	
Grade 6 to Ordinary Level Exam	231 (40.5)	46 (24.2)	95 (49.5)	91 (47.9)	
Passed Ordinary Level/Beyond	184 (32.3)	110 (57.9)	31 (16.3)	43 (22.6)	
Dietary Intake (Fruit and Vegetable)					
Less than 3 portions	304 (53.3)	71 (37.4)	134 (70.5)	99 (52.1)	< 0.001
More than 3 portions	266 (46.7)	119 (62.6)	56 (29.5)	91 (47.9)	
Migration Experience					
Number of Displacements					
Never/once	321 (56.3)	124 (65.6)	67 (35.6)	130 (68.4)	< 0.001
2 times	121 (21.2)	37 (19.6)	33 (17.5)	51 (26.8)	
More than 3 times	125 (21.9)	28 (14.8)	88 (46.8)	9 (4.7)	
Missing	3 (0.5)				
Duration of Stay in Refugee Camp					
Never	169 (29.7)	167 (87.9)	0 (0)	2 (1.1)	< 0.001
0–5 years	274 (48.1)	16 (8.4)	190 (100.0)	68 (35.8)	
More than 6 years	127 (22.3)	7 (3.7)	0 (0)	120 (63.2)	
Health Care Access					
Measles Vaccination					
Yes	307 (54.0)	108 (56.8)	89 (46.8)	110 (58.2)	0.007
No	179 (31.5)	49 (25.8)	79 (41.6)	51 (27.0)	
Don’t Know	83 (14.6)	33 (17.4)	22 (11.6)	28 (14.8)	
Outpatient Visits (last 3 mo.)					
Yes	231 (40.5)	75 (39.5)	100 (52.6)	56 (29.5)	< 0.001
No	339 (59.5)	115 (60.5)	90 (47.4)	134 (70.5)	
Social Environment					
Aware of Suicide					
Yes	238 (41.8)	53 (28.0)	60 (31.6)	125 (65.8)	< 0.001
No	332 (58.2)	137 (72.1)	130 (68.4)	65 (34.2)	
Aware of Violence Against Women					
Yes	117 (20.5)	32 (16.8)	19 (10.0)	66 (34.7)	< 0.001
No	453 (79.5)	158 (83.2)	171 (90.0)	124 (65.3)	
Aware of Violence Against Children					
Yes	76 (13.3)	14 (7.3)	16 (9.4)	46 (24.2)	< 0.001
No	494 (86.7)	176 (92.6)	174 (91.6)	144 (75.8)	
Aware of Alcoholism					
Yes	301 (52.8)	122 (64.2)	53 (27.9)	126 (66.3)	< 0.001
No	269 (47.2)	68 (35.8)	137 (72.1)	64 (33.7)	
Aware of Drug Addiction					
Yes	64 (11.2)	16 (8.4)	11 (5.8)	37 (19.5)	< 0.001
No	506 (88.8)	174 (91.6)	179 (94.2)	153 (80.5)	
Attempted Suicide					
Yes	7 (1.2)	4 (2.1)	3 (1.6)	0 (0)	0.153
No	563 (98.8)	186 (97.9)	187 (98.4)	190 (100.0)	
Experienced Domestic Violence					
Yes	2 (0.4)	0 (0)	1 (0.5)	1 (0.5)	0.605
No	568 (99.7)	190 (100.0)	189 (99.5)	189 (99.5)	
No	564 (99.0)	188 (99.0)	186 (97.9)	190 (100.0)	
Alcoholism					
Yes	5 (0.9)	3 (1.6)	0 (0)	2 (1.1)	0.244
No	565 (99.1)	187 (98.4)	190 (100.0)	188 (99.0)	
Smoking					
Yes	52 (9.1)	20 (10.5)	2 (1.1)	30 (15.8)	< 0.001
No	518 (90.9)	170 (89.5)	188 (99.0)	160 (15.8)	
Physical Environment					
Number of People at Home					
2 persons or less	93 (16.3)	18 (9.5)	46 (24.2)	29 (15.3)	< 0.001
3 persons	101 (17.8)	19 (10.1)	38 (20.0)	44 (23.2)	
4 persons	135 (23.7)	43 (22.8)	37 (19.5)	55 (29.0)	
5 or more	240 (42.2)	109 (57.7)	69 (36.3)	62 (32.6)	
Type of Wall					
Temporary material	202 (35.4)	2 (1.1)	187 (98.4)	13 (6.8)	< 0.001
Medium quality material	137 (24.0)	37 (19.5)	3 (1.6)	97 (51.1)	
High quality material	231 (40.5)	151 (79.5)	0 (0)	80 (42.1)	
Type of Roof					
Cadjan/palmyrah/other	103 (18.1)	16 (8.4)	18 (9.5)	69 (36.3)	< 0.001
Metal/Tar sheet	239 (41.9)	22 (11.6)	172 (90.5)	45 (23.7)	
Tile/asbestos	228 (40.0)	152 (80.0)	0 (0)	76 (40.0)	
Type of Floor					
Mud/dung	109 (19.1)	18 (9.5)	0 (0)	91 (47.9)	< 0.001
Terrazo/carpet/cement	461 (80.9)	172 (90.5)	190 (100.0)	99 (52.1)	
Source of Water					
Well without motor	156 (27.4)	49 (25.8)	0 (0)	107 (56.3)	< 0.001
Well with motor	162 (28.4)	117 (61.6)	0 (0)	45 (23.7)	
Water line	74 (13.0)	13 (6.84)	55 (29.0)	6 (3.2)	
Other^α^	178 (31.2)	11 (5.8)	135 (71.1)	32 (16.8)	
Ownership of Water					
Private	258 (45.3)	160 (84.2)	0 (0)	98 (51.6)	< 0.001
Public	312 (54.7)	30 (15.8)	190 (100.0)	92 (48.4)	
Type of Toilet					
Latrine and other	37 (6.5)	6 (3.2)	0 (0)	31 (16.3)	< 0.001
Water sealed pour flush	521 (91.4)	179 (94.2)	190 (100.0)	152 (80.0)	
Water sealed cistern flush	12 (2.1)	5 (2.63)	0 (0)	7 (3.7)	
Ownership of Toilet					
Private	289 (50.7)	159 (94.7)	0 (0)	110 (59.5)	< 0.001
Public	275 (48.3)	10 (5.3)	190 (100.0)	75 (40.5)	
Missing	6 (1.1)				
Cooking Fuel					
Gas	58 (10.2)	44 (23.2)	0 (0)	14 (7.4)	< 0.001
Kerosene	18 (3.2)	10 (5.3)	0 (0)	8 (4.2)	
Fire wood	489 (85.8)	131 (69.0)	190 (100.0)	168 (88.4)	
Other	5 (0.9)	5 (2.6)	0 (0)	0 (0)	

*HOC* Host community, *IDP* Internally Displaced Person, *RET* Returning refugee, *CI* Confidence Interval

^α^Other: tube well, river, tank, stream, and water browse

*p value from Pearson’s chi-squared test comparing variable with Displacement Status

Prevalence of mild depression was highest within the IDP group (10.5%) followed by HOC (5.8%) and RET (3.2%). The prevalence of having at least one chronic disease was 24.7% for HOC, 26.3% for IDP, and 24.2% for RET. The median and inter-quartile range (IQR) for SF-36 total score were 84 (IQR: 71–90) for HOC, 66 (IQR: 47–85) for IDP, and 85 (IQR: 71–90) for RET. Variables that had a significant univariable association with each outcome are listed in Table 2.

**Table 2:** Covariates found to be significantly associated with each outcome in the univariable analysis

	Patient Health Questionnaire 9 (PHQ-9)	Chronic Disease Status	Short Form Questionnaire 36 (SF-36) Total
Primary Exposure			Displacement status**
Domain 1: Predisposing Factors	Sex**	Age**	Sex**
	Age **	Debt**	Age**
	Family Income**	Education**	Family Income**
	Employment Status**	Dietary Intake**	Debt**
		Ethnicity*	Employment Status**
			Education**
			Dietary Intake**
			Marital Status*
Domain 2: Migration Experience			Number of Displacements**
			Duration in Refugee Camp**
Domain 3: Health Care Access	Measles Vaccination*	Measles Vaccination**	Measles Vaccination**
	Outpatient Visits**	Outpatient Visits**	Outpatient Visits**
Domain 4: Social Environment	Attempted Suicide**	Aware Violence Against Women**	Aware Suicides**
	Aware of Alcoholism*	Aware Violence Against Children*	Aware Violence Against Women**
		Aware Alcoholism*	Aware Alcoholism**
		Attempted Suicide**	Aware Drug Addiction*
		Alcoholism*	
Domain 5: Physical Environment	Wall**	Number of People at Home**	Number of People at Home**
	Roof**	Type of Toilet*	Type of Wall**
	Floor**		Type of Roof**
	Source of Water*		Type of Floor**
	Ownership of Toilet*		Source of Water**
			Type of Toilet**
			Cooking Fuel**

*p value < 0.15

**p value < 0.05

The IDP group had a 6.7-point lower SF-36 perceived quality of life score than HOC and RET, after adjusting for other variables (Table 3). Female gender, increasing age, lower educational attainment, unemployment, lower income, being in debt, low dietary diversity, higher number of displacements, uncertain measles vaccination status, and awareness of alcohol and drug addiction among family members or others were all associated with lower quality of life. No association was shown between displacement status and mental health status or chronic disease status. Male gender, increasing age, high dietary diversity, and awareness of alcohol addiction among family members or others were all associated with higher chronic disease status (Table 4). Female gender, increasing age, and unemployment were all associated with higher risk of depression (Table 5).

**Table 3:** Fully adjusted generalised linear regression model for the Short Form Questionnaire 36 total score

	β Coefficient (95% CI)	*P* value
Displacement Status		
HOC	Reference	
IDP	−6.7 (−11.4, −2.0)	0.006*
RET	4.0 (− 1.0, 8.9)	0.114
Predisposing Factors		
Sex		
Male	Reference	
Female	−3.6 (−7.1, −0.1)	0.042*
Age		
< 29	Reference	
30–39	−2.9 (− 6.2, 0.3)	0.078
40–49	− 5.9 (− 9.5, − 2.3)	0.001*
50–59	− 9.6 (− 13.7, − 5.6)	< 0.001*
> =60	− 18.2 (− 23.1, − 13.3)	< 0.001*
Education		
No school	Reference	
Grade 1–5	1.8 (− 3.5, 7.1)	0.504
Grade 6 to O/L	3.2 (−2.0, 8.4)	0.222
Passed O/L and beyond	9.0 (3.4, 14.6)	0.002*
Monthly Family Income		
< 5000	Reference	
5000–9999	4.1 (0.8, 7.4)	0.015*
> =10,000	3.5 (−0.3, 7.3)	0.072
Employment Status		
Yes	Reference	
No	−3.4 (−7.1, 0.2)	0.065
Debt		
No	Reference	
Yes	- 5.6 (− 8.5, −2.7)	< 0.001*
Dietary Intake		
More than 3 portions	Reference	
Less than or equal to 3 portions	−5.1 (− 7.6, − 2.5)	< 0.001*
Migration Experience		
Number of Displacements		
Never / One time	Reference	
2 times	−1.6 (−4.6, 1.4)	0.290
3 or more times	−3.7 (−7.0, − 0.5)	0.022*
Health Care Access		
Measles Vaccination		
Yes	Reference	
No	− 1.4 (− 4.3, 1.4)	0.330
Don’t Know	−6.0 (− 9.5, − 2.5)	0.001*
Social Environment		
Aware of Alcoholism		
No	Reference	
Yes	1.6 (−2.8, 6.0)	0.469
Alcoholism * Displacement Status (Interaction terms)		
No * HOC	Reference	
Yes * IDP	11.3 (5.1, 17.5)	< 0.001*
Yes * REF	1.2 (− 4.8. 7.2)	0.698
Aware of Drug Addition		
No	Reference	
Yes	−8.8 (−12.7, − 4.9)	< 0.001*
Physical Environment		
Number of People at Home		
2 persons or less	Reference	
3 persons	−3.9 (− 8.1, 0.3)	0.065
4 persons	−1.1 (− 5.1, 2.9)	0.588
5 or more	0.1 (−3.8, 3.9)	0.979

*HOC* Host community, *IDP* Internally Displaced Person, *RET* Returning refugee, *CI* Confidence Interval, *O/L* Ordinary Level

*p value < 0.05

**Table 4:** Fully adjusted multivariable logistic regression model for Chronic Disease Status

	Adjusted OR (95% CI)	*P* value
Displacement Status		
HOC (Ref)	1	
IDP	1.3 (0.4, 4.4)	0.63
RET	0.5 (0.2, 1.1)	0.103
Predisposing Factors		
Sex		
Men (Ref)	1	
Female	0.5 (0.2, 1.0)	0.055
Sex * Displacement Status (Interaction Terms)		
Men + HOC (Ref)	1	
Women + IDP	1.4 (0.4, 5.2)	0.608
Women + RET	3.9 (1.3, 11.1)	0.012*
Age		
< 29 (Ref)	1	
30–39	7.2 (3.3, 17.1)	< 0.001*
40–49	8.6 (3.5, 21.0)	< 0.001*
50–59	17.4 (7.0, 43.1)	< 0.001*
> =60	23.0 (8.8, 59.9)	< 0.001*
Dietary Intake (Fruit and Veg)		
Less than 3 portions (Ref)	1	
More than 3 portions	2.1 (1.3, 3.2)	0.001*
Social Environment		
Aware of Alcoholism		
No (Ref)	1	
Yes	2.0 (1.2, 3.2)	0.005*
Attempted Suicide		
No (Ref)	1	
Yes	11.0 (1.8, 68.0)	0.011*
Aware of Violence against Children		
No (Ref)	1	
Yes	0.5 (0.3, 1.1)	0.067

*HOC* Host community, *IDP* Internally Displaced Person, *RET* Returning Refugee, *OR* Odds Ratio, *CI* Confidence Interval

*p value < 0.05

**Table 5:** Fully adjusted multivariable logistic regression model for depression status, as determined through the Patient Health Questionnaire 9 (PHQ-9)

PHQ-9	Adjusted OR (95% CI)	*P* value
Displacement Status		
HOC (Ref)	1	
IDP	1.1 (0.5, 2.5)	0.791
RET	0.6 (0.2, 1.7)	0.320
Predisposing Factors		
Sex		
Male (Ref)	1	
Female	3.2 (0.8, 12.5)	0.094
Age		
< 29 (Ref)	1	
30–39	6.3 (1.4, 28.6)	0.018*
40–49	3.1 (0.6, 16.3)	0.187
50–59	5.2 (1.0, 26.6)	0.048*
> =60	14.9 (3.1, 71.9)	0.001*
Employed		
Yes (Ref)	1	
No	5.6 (0.7, 48.5)	0.118

*HOC* Host community, *IDP* Internally Displaced Person, *RET* Returning Refugee, *OR* Odds Ratio, *CI* Confidence Interval

*p value < 0.05

## Discussion

### Mental health status

We found that the IDP group had almost a double risk of mild depression than HOC and RET, although the association was weak. Similar studies in post-conflict settings detected higher prevalence of depression among IDPs, e.g. 67% in Afghanistan and 22% in Ukraine [26, 27]. Studies conducted by Somasundaram and colleagues in Sri Lanka have explored the effect of ‘collective trauma’ caused by the conflict’s disruption of traditional family and community networks on the mental health of affected communities. [28] This may be a feature in the mental health status of the study population. Once adjusted for potential confounders, the overall association between displacement status and depression did not vary remarkably between the three groups. We identified other factors attributable to a higher risk of developing depression. Older age remained strongly associated with depression, with 60 years or older adults having almost 15 times the risk of depression (albeit with limited precision). This could reflect the different lengths of exposure to conflict among the various age groups, with older participants enduring longer exposure to conflict and its related trauma [29]. Female sex was weakly associated with 3 times the risk of depression. As found in the literature, women in conflict settings are often at a higher risk of mental health disorders due to shifts in traditional gender dynamics and gender-based violence [30]. Our findings are consistent with previous studies on depression conducted within the post-conflict setting in Sri Lanka [15, 23, 29, 31, 32].

### Chronic disease status

Conflicts are increasingly affecting countries with higher incomes and longer life expectancies, resulting in conflict-affected and displaced populations experiencing an elevated burden of chronic diseases [4, 5]. Even during the conflict, although not uniformly across the country, Sri Lanka’s life expectancy increased (68 years in 1982 to 72 years in 2009) while overall fertility rate dropped (4.3 in 1970 to 2.3 in 2012), leading to population aging and a higher prevalence of NCDs [33–35].

In general, women were found to have half the risk of chronic disease. There was significant interaction between sex and displacement status: RET women had almost 4 times the risk of chronic disease when compared to HOC men. This finding might be attributable to RET women potentially having greater access to health services in India compared to HOC and IDP women in Sri Lanka and thus a higher probability of receiving a chronic disease diagnosis. Additionally, in most contexts, women utilise health care services more than men, which could help explain the difference between RET men and women. However, further research is required for confirmation. As expected, there is very strong evidence of a linear trend between the risk of having at least one chronic disease and increasing age. Participants with a higher fruit and vegetable intake (more than 3 portions a day) have two times higher risk of having a chronic disease. Eating a more diverse diet in itself reduces the risk for cardiovascular diseases and some cancers [36]; however, in this case, it could be interpreted as a proxy for higher income and potentially having the means to buy and consume foods with higher fat and salt content. This could explain the observed relationship.

Being aware of alcoholism and alcohol abuse is associated with almost double the risk of chronic disease. Alcohol has been seen in the literature as a coping strategy for the stressors related to forced displacement as well as a major risk factor for NCDs [36, 37]. Previous studies have found that IDP who do drink alcohol are more likely to have an alcohol use disorder [38]. The high prevalence of perceived alcohol abuse in the HOC and RET groups (64% in HOC and 66% in RET) suggests alcohol as an important determinant of chronic disease. Further research needs to examine the behavioural risk factors and underlying metabolic or physiological causes of chronic diseases in displaced populations.

### Quality of life

Median SF-36 total scores were relatively high when compared to other conflict-affected populations, e.g. in the 40s in post-conflict southern Sudan and Uganda [39, 40]. Again, this could be due to the prolonged nature of the Sri Lankan conflict and its effect on mental health as described above. Female sex and older age were associated with lower general physical and mental health. This finding is consistent with similar studies of displaced populations in post-conflict southern Sudan, Uganda, and Ethiopia [39–41]. Low income, being in debt, and limited fruit and vegetable consumption were also associated with lower quality of life. This could be attributable to stress, uncertainty and food insecurity [30, 40]. Having been displaced three or more times was negatively associated with quality of life. Roberts et al. found a similar association between number of displacements (displaced more than once) and reduced general physical health in southern Sudan [39]. When people are forced to endure repeated episodes of displacement, there is a higher chance of experiencing adversity or trauma related to the displacement process that can accumulate to negatively affect their well-being.

Strong evidence of an interaction between awareness of alcohol abuse and displacement status was observed: IDPs who were aware of alcohol abuse in their community scored higher on the quality of life survey. However, IDP had the lowest prevalence of awareness of alcohol abuse (28%), almost half compared to HOC and RET. IDP who are aware of alcoholism in their community may preferentially socialise with other IDPs who drink and can afford to buy alcohol. This awareness could reflect having greater disposable income, which was positively associated with better overall health. Further research is required to confirm this interaction and its implications.

### Limitations of the study

The target population of this study was restricted to Vavuniya district thus limits the generalisability of the findings. Using cross-sectional data, we are unable to fully infer causal associations between displacement status or the other risk factors and any of the measured outcomes. Moreover, cross-sectional surveys rely upon self-reporting of past events, which can be influenced by recall bias. While the SF-36 and PHQ-9 surveys have both been widely used and validated, there is still potential for misclassification due to the present state effect, a type of recall bias whereby participants use information on their current state to recreate their former state, causing over- or under-estimation of the outcome measure [42]*.* Chronic disease status was determined through self-reporting of a previous diagnosis. This could be subject to potential misclassification if the participant had a previously undiagnosed chronic disease (i.e. Katulanda et al. found that one-third of people in Sri Lanka with diabetes are undiagnosed) or did not recall their chronic disease at the time of the interview [43]. Moreover, the low prevalence of outcomes explored in the social environment domain such as suicide ideation and experience of violence indicate that these topics are difficult to discuss due to stigma and sensitivities within the culture, despite having medically trained persons involved in the data collection.

## Conclusion

Our findings showed that displacement experience alone is not a successful predictor of depression or chronic disease status, but is strongly associated with quality of life. Even so, the results suggest that understanding the health effects of displacement requires an assessment of multiple levels of risk factors, from the most distal pre-disposing factors to most proximate physical environment factors. The findings of this study revealed an important need to provide adequate mental health care to conflict-affected persons and those who returning to their areas of origin after prolonged displacement. Whilst boosting primary care services is a critical component, recent pilot interventions in northern Sri Lanka such as treatment and referral of patients with common mental disorders via the World Health Organization Mental Health Gap Action Programme (mhGAP) hold promise [44]. This should be replicated in other post-conflict settings and expanded to include other NCDs, thus further closing service delivery gaps and strengthening health system capacities.

## Abbreviations

95% CI: 95% Confidence Interval
HOC: Host Community
IDP: Internally Displaced Persons
IQR: Interquartile Range
LRT: Likelihood Ratio Test
NCD: Non-Communicable Diseases
OR: Odds Ratio
PHQ-9: 9-item Patient Health Questionnaire
RET: Returning Refugees
RR: Rate Ratio
Rs.: Sri Lankan Rupees
SF-36: 36-item Short Form Questionnaire
